# Anti-Inflammatory Effects of Acupuncture Stimulation via the Vagus Nerve

**DOI:** 10.1371/journal.pone.0151882

**Published:** 2016-03-18

**Authors:** Hee-Don Lim, Min-Hee Kim, Chan-Yong Lee, Uk Namgung

**Affiliations:** 1 Department of Oriental Medicine, Daejeon University, Daejeon, Republic of Korea; 2 Department of Microbiology and Biotechnology, Daejeon University, Daejeon, Republic of Korea; University of Louisville, UNITED STATES

## Abstract

Although acupuncture therapy is widely used in traditional Asian medicine for the treatment of diverse internal organ disorders, its underlying biological mechanisms are largely unknown. Here, we investigated the functional involvement of acupuncture stimulation (AS) in the regulation of inflammatory responses. TNF-α production in mouse serum, which was induced by lipopolysaccharide (LPS) administration, was decreased by manual acupuncture (MAC) at the zusanli acupoint (stomach36, ST36). In the spleen, TNF-α mRNA and protein levels were also downregulated by MAC and were recovered by using a splenic neurectomy and a vagotomy. c-Fos, which was induced in the nucleus tractus solitarius (NTS) and dorsal motor nucleus of the vagus nerve (DMV) by LPS and electroacupuncture (EAC), was further increased by focal administration of the AMPA receptor blocker CNQX and the purinergic receptor antagonist PPADS. TNF-α levels in the spleen were decreased by CNQX and PPADS treatments, implying the involvement of inhibitory neuronal activity in the DVC. In unanesthetized animals, both MAC and EAC generated c-Fos induction in the DVC neurons. However, MAC, but not EAC, was effective in decreasing splenic TNF-α production. These results suggest that the therapeutic effects of acupuncture may be mediated through vagal modulation of inflammatory responses in internal organs.

## Introduction

Acupuncture therapy has been used to treat diverse disorders of the internal organ in several Asian countries, and its use is increasing in Western countries as well [1]. According to the principles of traditional Asian medicine, stimulation at an acupuncture point (acupoint) refurbishes the flow of qi, a vital force, through the energy channel (meridian) among the organs in different parts of the body; nevertheless, the biological basis for how acupuncture stimulation (AS) is linked to the pathogenesis of internal organ is still unknown.

Among several biological correlates that may explain the principle of acupuncture, the autonomic nervous system (ANS) is frequently considered to be a mediator of AS because it can interconnect external somatosensory inputs with internal organ responses via the central neural networks [2,3]. Of the sympathetic and the parasympathetic nerves comprising the ANS, the vagus nerve, which broadly regulates the functions of internal organs, has been a primary target for exploring the possible effect of cutaneous AS on internal organs [4–7]. For instance, stimulation at the lower limb acupoint ST36 has been shown to induce c-Fos protein signals in the nucleus tractus solitarius (NTS), the dorsal vagal complex (DVC) area receiving a primarily visceral afferent and having bidirectional connections to the areas of the upper brain [8,9]. In addition, AS at the abdomen acupoint ST25 has been shown to induce c-Fos signals in neurons at the rostroventrolateral medulla (RVLM), a brain center that sends out axons to the spinal cord for sympathetic regulation of cardiovascular functions [10]. Interestingly, some RVLM neurons have been observed to respond to electrical stimulations on both the vagus nerve and the sciatic nerve [11]. While these and other reports imply that the signals from diverse acupoints may be transmitted into the vagus nerve and affect the function of ANS, systematic studies on the neuronal circuits in the brainstem, which connect somatosensory afferents and the vagal efferent, have not been reported so far.

Vagus nerve stimulation (VNS) has been demonstrated to reduce inflammatory responses in animals given lipopolysaccharide (LPS) [12,13]. In a series of reports, Tracey and colleagues delineated VNS-mediated adrenergic inputs to T lymphocytes and inhibition of tumor necrosis factor-α (TNF-α) production through α7 nicotinic receptor activation in LPS-activated splenocytes [14]. Here, we hypothesize that if AS signals are transmitted to the DVC and activate vagal efferents, changes in inflammatory responses might occur in internal target organs such as in the spleen. Indeed, our results show that AS on ST36 alters the levels of TNF-α production in the spleen as well as the serum. Our immunohistochemical data further indicate that neural circuits in the DVC are involved in mediating AS signals into the vagus nerves.

## Materials and Methods

### Animals

Balb/c mice (8- to 12-weeks old male, 20–25 g, Dae Han Biolink, Eumseong, Korea) were maintained in an animal room with regulated temperature (22°C), 60% humidity, and a 12-hour light/12-hour dark cycle. Animals were allowed to eat commercial chow and to drink water ad libitum. This study was carried out in strict accordance with the recommendations in the Guide for the Care and Use of Laboratory Animals of the National Institutes of Health. All protocols involving preopertative, operative, and postoperative animal care were approved by the Daejeon University Institutional Animal Use and Care Committee and were in accordance with the Animal-use Statement and Ethics Committee Approval Statement for Animal Experiments provided by Daejeon University (Daejeon, Korea). None of animals became ill and died during the study. Postoperative animals were euthanized by overdose of ketamine and zylazine (80 mg/kg of ketamine and 5 mg/kg of xylazine). All surgical procedures were conducted in a room under well-ventilated, sterile conditions, and all efforts were made to minimize suffering.

### Endotoxemia

Endotoxemia was induced in the mice by administering LPS from *Escherichia coli* 0127:B8 (Sigma-Aldrich, St. Louis, USA) intraperitoneally (60 μg/kg or 15 mg/kg). Blood was sampled from the tail at 30, 60, 90, 120 and 180 minutes after LPS administration.

### Acupuncture stimulation

For manual acupuncture (MAC) under anesthesia, mice were administered 80 mg/kg of ketamine and 5 mg/kg of xylazine, and the needle (0.20 × 7 mm, HL Medical, Seoul, Korea) was inserted bilaterally into the zusanli acupoint (ST36; 3–4 mm below the knee midline and laterally 1–2 mm at a depth of 2–3 mm). Acupuncture needles were inserted for 30 minutes with a slow rotation every 5 minutes. For electroacupuncture (EAC) at ST36, electric stimulation was given at 1 volt, 1 Hz, with a 2-msec pulse duration for 30 min by using an isolated pulse stimulator (A-M System 2100, Carlsborg, USA). For the acupuncture experiment using unanesthetized animals, mice were placed and immobilized in 50-ml conical tubes and AS was given in the same way as it was for the anesthetized animals.

### Enzyme-linked immunosorbent assay (ELISA) for TNF-α measurement

Blood was centrifuged at 800 g for 10 minutes, and the supernatants were collected for TNF-α analysis in plasma. ELISA was performed as recommended by the manufacturer (mouse TNF-α ELISA, eBioscience Inc, San Diego, USA). Briefly, to a 96 well plate pretreated with coating buffer was added 100 μl of capture antibody (anti-mouse TNF-α) in coating buffer. The plate was sealed and incubated overnight at 4°C. Wells were aspirated and washed 5 times with 200 μl of a washing buffer (0.05% Tween-20 in phosphate buffered saline (PBS)) and were blocked with 200 μl of assay diluent solution (10% fetal bovine serum in PBS). After incubation at room temperature for 1 hour, 100 μl of standard recombinant mouse TNF-α along with the samples were added to the appropriate wells, and the wells were incubated at room temperature for 2 h. Then, 100 μl of detection antibody (anti-mouse TNF-α) in assay diluents were added to each well, and the wells were incubated at room temperature for 1 h. Finally, 100 μl of avidin-horseradish peroxidase (HRP) diluted in assay diluents were added, and the wells were incubated at room temperature for 30 min to activate the substrate solution. The reaction was stopped by using 2N H_2_SO_4_. Values of the optical density of the samples were read at 450 nm by using a spectrophotometer (Thermo Max Microplate Reader, Molecular Devices LLC, Sunnyvale, USA).

### Reverse transcription-polymerase chain reaction (RT-PCR) and real-time PCR

Total RNA was isolated from the spleen by using Easy-BLUE reagent (Intron, Sungnam, Korea). A reaction for cDNA synthesis was carried out in 30 μl using total RNA (1 μg) as a template, 1X reaction buffer (50 mM Tris-HCl, 75 mM KCl, 3 mM MgCl2, 10 mM DTT)), 104 μM dNTP), RNasin (30 U), random primer (16 μM Promega), MMLV reverse transcriptase (200 U, Promega, Madison, USA), for 2 h at 37°C. Real-time PCR was performed as follows. Synthesized cDNA (300 ng), 1X Power SYBR Green PCR Master mix (AmpliTaq Gold DNA Polymerase, SYBR Green I Dye, dNTP mix, optimized buffer), 0.15 μM of forward and reverse TNF-alpha primers, and PCR-grade water in a 20 μl of reaction volume were added to each well of 96-well plate (Applied Biosystems, MicroAmp Optical 96-well Reaction plate). For the synthesis of a mouse GAPDH reference gene, cDNA (300 ng), 1x TaqMan Gene Expression Master Mix (AmpliTaq Gold DNA Polymerase, ROX^™^ dye, dNTP mix, optimized buffer), and 1X mouse GAPDH primer (Applied Biosystems, Mouse GAPD (GAPDH), endogenous control (VIC^®^/MGB probe primer), and PCR = grade water were mixed in a 20 μl of reaction volume. Plate was covered with a film (Applied Biosystems, MicroAmp Optical Adhesive Film) and was centrifuged at 2,900 rpm (BECKMAN, GS-6R Centrifuge) for 5 min to spin down samples. PCR was carried out using an instrument (Applied Biosystems, 7500 Real Time PCR System) by activating Taq polymerase for 2 min at 50°C and for 10 min at 95°C, followed by 40 cycles with 15 s at 95°C (denaturation) and 1 min at 60°C (annealing). The results were shown as a ratio of TNF-α mRNA to GADPH reference mRNA. The primer sequences used for real-time PCR for TNF-α mRNA were the forward primer (5’-CATCTTCTCAAAATTCGAGTGACAA-3’) and the reverse primer (5’- TGGGAGTAGACAAGGTACAACCC-3’). Values of relative quantitation (RQ) of TNF-α mRNA expression relative to GAPDH reference mRNA was calculated by a software converting program (Applied Biosystems) for threshold cycle (Ct) data.

For RT-PCR analysis, 30 cycles of amplification were optimal for a quantitative comparison of the TNF-α mRNA expression among the samples. The primer sequences used for PCR were the forward primer (5’-TCAGCCTCTTCTCATTCCTG-3’) and the reverse primer (5’-CAGGTACATGGGCTCATACC-3’) for TNF-α mRNA, and the forward primer (5’-CACACTGTGCCCATCTATGA-3’) and the reverse primer (5’-TACGGATGTCAACGTCACAC-3’) for actin mRNA. Amplified DNA sizes were 497 bp and 409 bp for TNF-α and actin, respectively. PCR-amplified DNA was analyzed on agarose gels, and the band intensities from the captured images were quantified using i-Solution software (Image & Microscope Technology, Burnaby, Canada).

### Immunofluorescence staining

Spleen and brain tissues were embedded and frozen at –20°C. Sections (15 or 20 μm thickness) were cut on a cryostat, mounted on positively charged slides, and subjected to immunostaining as described previously [15]. Briefly, sections were fixed with 4% paraformaldehyde and 4% sucrose in PBS at room temperature for 45 min, were permeabilized with 0.5% Nonidet P-40 in PBS at room temperature for 30 min, and were blocked with 5% goat serum, 5% horse serum and 3% bovine serum albumin in PBS and 0.1% triton X-100 (PBST) for 4 h at room temperature. Sections were incubated 4 h at room temperature with primary antibodies, and washed three times 10 min each with PBST. Sections were incubated with secondary antibodies for 2 h at room temperature in a dark room and washed three times with PBST. Cellular nuclei were stained with Hoechst 33258 pentahydrate (25 μg/ml, bis-benzimide; Sigma-Aldrich) for 10 min before the final wash with 0.1% Triton X-100 in PBS. Sections were then coverslipped with gelatin mounting medium. Samples were viewed with a Nikon fluorescence microscope, and images were captured using a Nikon camera (Nikon, Tokyo, Japan). The merged images were produced using the layer-blending mode options of Adobe Photoshop. Primary and secondary antibodies were diluted in blocking solution. Primary antibodies used in the present study were goat polyclonal anti-TNF-α (Santa Cruz Biotech.; 1:400) and rabbit polyclonal anti-TNF-α (Abcam, Cambridge, England;1:400), rabbit polyclonal anti-P2X2 (Thermo Scientific, Rockford, USA, 1:400), rabbit polyclonal anti-GluR2/3 (Millipore, Darmstadt, Germany; 1:200), mouse monoclonal and rabbit polyclonal anti-c-Fos (Santa Cruz Biotech.; 1:200 and 1:400 respectively), and mouse monoclonal N52 anti-neurofilament 200 (Sigma-Aldrich; 1:400). Secondary antibodies were fluorescein-goat anti-mouse IgG antibody (H+L) (Invitrogen, Eugene, USA; 1:400), Alexa fluor 488 donkey anti-goat IgG antibody (Invitrogen, Eugene, USA; 1:400,) and rhodamine goat anti-rabbit antibody (Invitrogen; 1:400).

For quantification of the TNF-α signal from spleen tissues, the fluorescence images in the slide were captured by fluorescence microscope coupled with digital camera and transferred to the i-Solution software program (Image and Microscope Technology). Digital images for analysis were taken under the same exposure conditions among experimental groups and no modifications in the brightness and contrast were made for the numerical measures of pixel intensity. Three to five microscopic fields (field size: 410 x 330 mm) were randomly taken from each section and the average value from 4 nonconsecutive sections per each animal was used for statistical comparison among experimental groups. For the quantification of c-Fos signals in the NTS and DMV areas in the horizontal brain sections, digital images captured from the fluorescence microscope under the same exposure conditions were transferred to Adobe Photoshop software. Boundaries of NTS and DMV areas were identified by Hoechst nuclear counterstaining and marked on the images, and individual c-Fos signals which were colocalized with Hoechst-stained nuclei as clear spots were counted manually in each field (410 x 330 mm). The number of average signals from 4 nonconsecutive sections per each animal was compared among experimental groups.

### Brainstem drug injection, splenic neurectomy and vagotomy

Mice were anesthetized with ketamine and xylazine. CNQX (6-Cyano-7-nitroquinoxaline-2,3-dione, disodium salt hydrate, Sigma-Aldrich) and PPADS (pyridoxalphosphate-6-azophenyl-2',4'-disulfonic acid, Sigma-Aldrich) were dissolved in artificial cerebrospinal fluid (ACSF; 126 mM NaCl, 2.5 mM KCl, 1.25 mM NaH_2_PO_4_, 2 mM CaCl_2_, 2 mM MgCl_2_, 26 mM NaHCO_3_, 10 mM D-glucose). One microliter of CNQX (1 mg/ml in ACSF), or PPADS (1 mg/ml), or a mixture consisting of 0.5 μl of CNQX and 0.5 μl of PPADS was injected into the DVC (-7.2 mm from the bregma, +0.6 mm from the midline, -5.0 mm dorsoventral from the surface of the brain) for 3 min by using a picoinjector (Harvard Apparatus PLI-100, Holliston, USA). Histological examinations of brain sections by using hemotoxylin and eosin staining did not show any abnormalities in drug-treated animals compared to non-treated controls (data not shown). Drug injection was carried out 60 min after endotoxemia, and was immediately followed by AS.

For the splenic neurectomy, the mice were anesthetized with 80 mg/kg of ketamine and 5 mg/kg of xylazine. An incision along the abdominal midline was used to expose the splenic nerve. Once identified and isolated, splenic nerves were cut using sharp scissors at a location close to the spleen. For mice undergoing a sham operation, the abdominal midline was cut, and splenic nerves were isolated, but no nerve neurectomy was performed. Animals were allowed to recover for 7 days before inducing endotoxemia and applying AS. A vagotomy was performed in the mice anesthetized with ketamine and xylazine. A left vagus nerve was exposed on a cervical midline and excised. Animals were used for acupuncture experiments 7 days later.

### Statistical analysis

Data were presented as standard error of mean (SEM). The means of the data in individual groups were compared by using the Student’s t-test or the one-way or two-way ANOVA, with Tukey’s post-hoc tests for multiple comparisons (SPSS computer software version 12.0). Statistically significant differences were reported as *p < 0.05, **p < 0.01, or ***p < 0.001.

## Results

**Fig 1:**
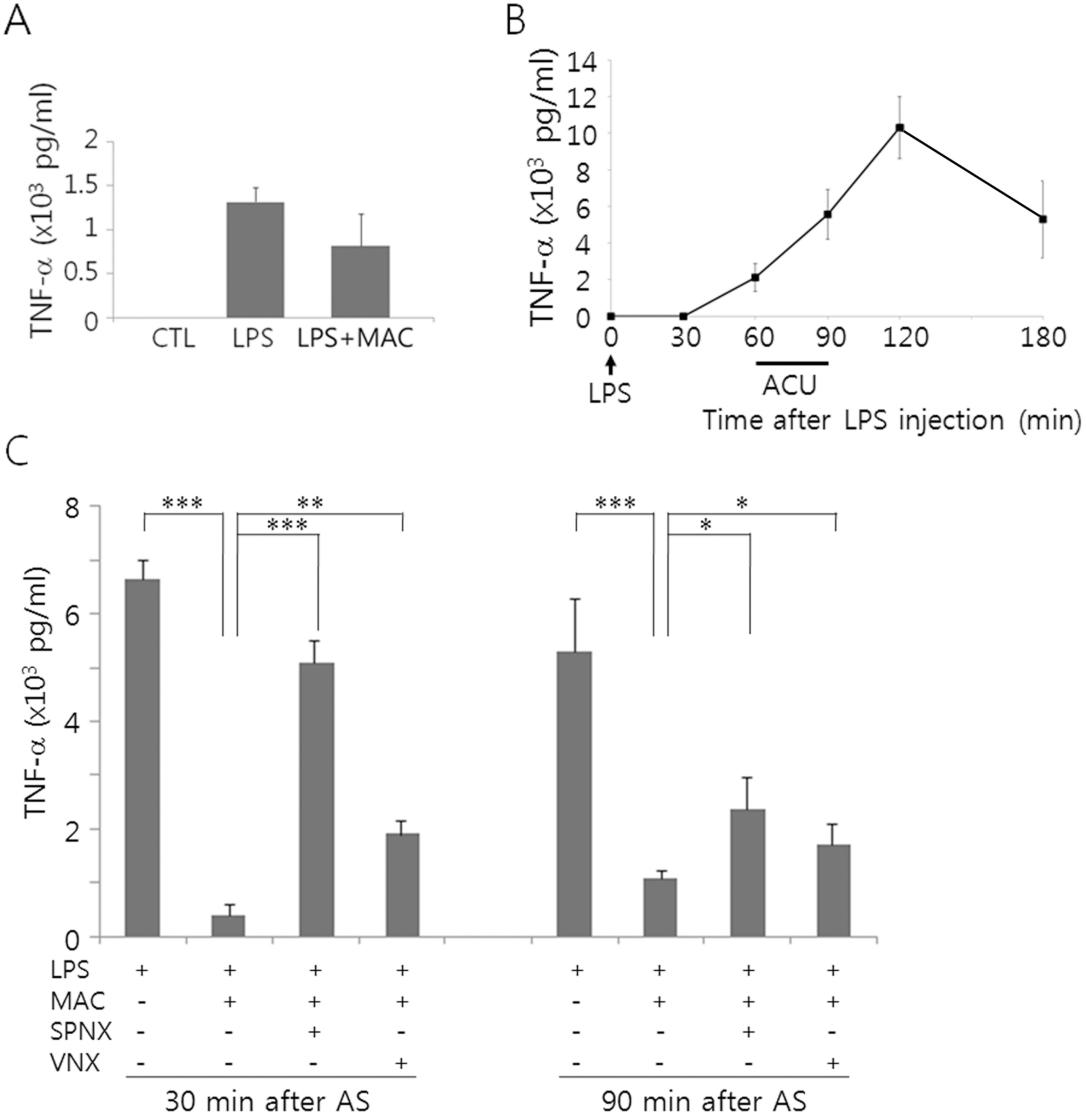
Profile of TNF-α production in the serum following AS in mice. (A) Comparison of TNF-α levels among the non-treated control (CTL), LPS (60 μg/kg), and the LPS plus MAC. MAC stimulation was given 60 min after LPS administration for 30 min, and TNF-α levels in the serum were compared 30 min after the acupuncture. (B) Time-dependent changes of TNF-α in the mice injected with LPS (15 mg/kg). (C) Comparison of TNF-α levels among animal groups with different treatments as indicated in the Figure. Data are means ± SEM [number of animals in each group = 3 in (A) and 4 in (B and C)]. *p<0.01, **p<0.01, ***p<0.001 (one-way ANOVA).

To examine the regulatory effects of AS on serum TNF-α production, we applied MAC for 30 min on the ST36 acupoint 60 min after LPS, and determined serum TNF-α 30 min or 90 min after the termination of AS. We initially tried to inject with 60 μg/kg of LPS, a dose which has been used to induce TNF-α in rats [16]. In our experimental condition using mice, TNF-α levels, when measured 30 min after AS, were much lower than the values obtained from the experiments using rats [16] and were highly variable among individual animals (Fig 1A). At the time point of 90 min after AS, TNF-α levels were further decreased and undetectable in some animals (data not shown). Thus, we attempted a higher dose (15 mg/kg), as has been used from the previous studies for the induction of TNF-α in mouse serum [17]. Mice given LPS with 15 mg/kg showed time-dependent changes in TNF-α with the peak at 120 min after LPS injection and maintained high level up to 3 h (Fig 1B). We adapted this dose of LPS because we sought to define the regulatory effects of AS on serum TNF-α, whose induction lasted at least several hours. TNF-α levels were significantly decreased compared to the LPS controls at 30 min and 90 min after AS (Fig 1C).

**Fig 2:**
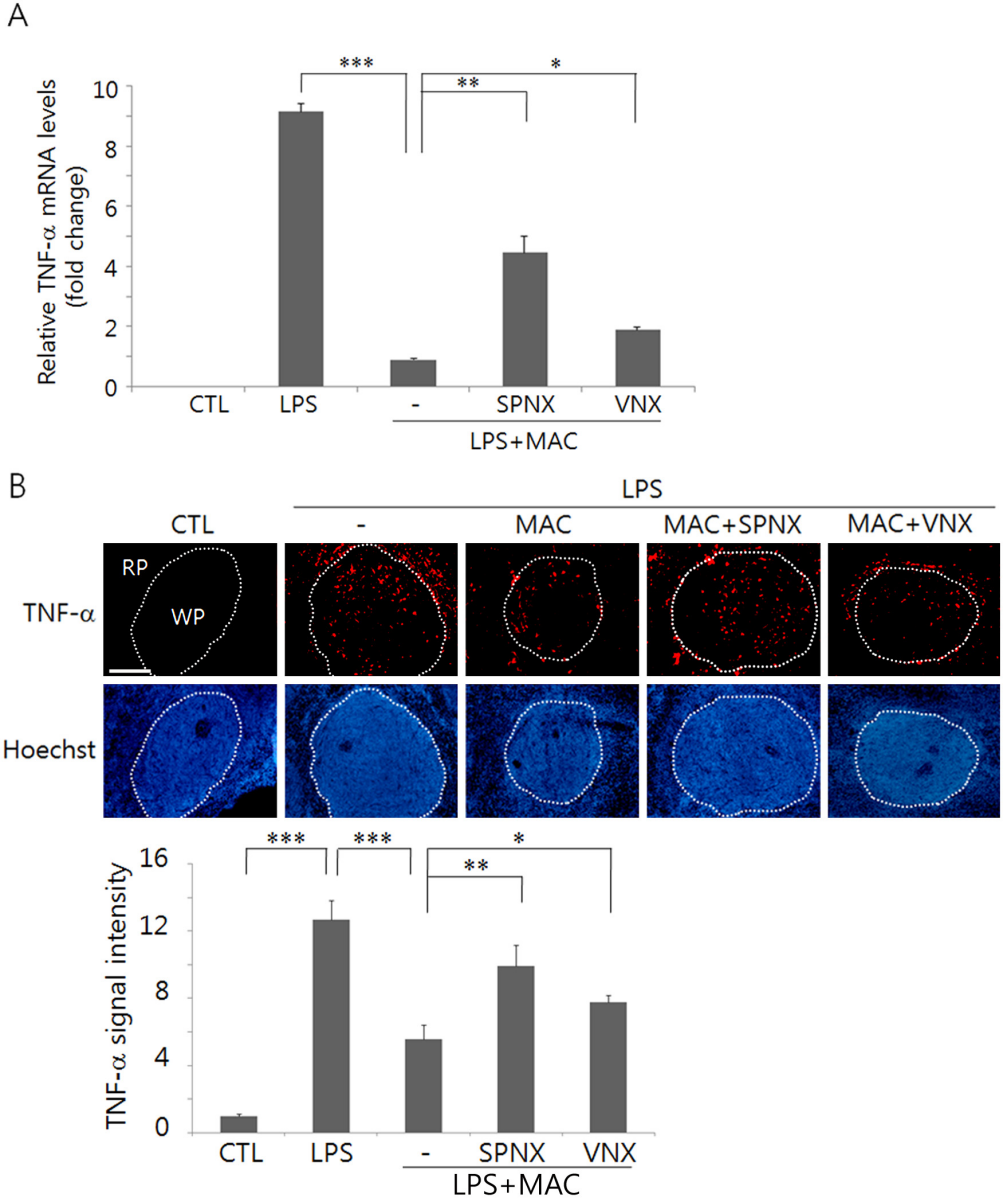
Regulation of TNF-α production by using MAC and a splenic neurectomy (SPNX) and a vagotomy (VNX). (A) A real-time PCR analysis for TNF-α mRNA expression in the spleen. Graph shows the fold changes of TNF-α mRNA levels in each group relative to internal loading control GAPDH mRNA. (B) Changes in the TNF-α protein signals due to MAC, SPNX, and VNX. The upper pictures are the representative images, and the lower graph shows quantitation of the protein signals in the field. (WP: white pulp, RP: red pulp). Spleen tissues were prepared from animals that had been sacrificed 90 min after AS and were used for real-time PCR and immunofluorescence analyses. *p<0.05, **p<0.01, ***p<0.001 (one-way ANOVA in A and B; n = 4 independent experiments). CTL shows the non-treated controls. The scale bar in (B) is 100 μm.

Macrophages in the spleen are the major source of inflammatory cytokines following LPS administration. Thus, we determined TNF-α production in the spleens of the animals given LPS and MAC, and investigated whether neural inputs to spleen were required for the regulation of TNF-α production. A splenic neurectomy abrogated efficiently the inhibition of TNF-α production in the serum by acupuncture when measured 30 min after AS. A vagotomy also elevated TNF-α levels, but less effective to eliminate the inhibitory effects of AS on TNF-α production (Fig 1C). Then we examined whether AS affected TNF-α production in the spleen. Real-time PCR analysis showed that TNF-α mRNA was highly induced in the spleen following LPS administration and was downregulated by MAC (Fig 2A). Then in animals given LPS and MAC, TNF-α mRNA levels were upregulated by a splenic neurectomy and were also elevated significantly by a vagotomy. Immunofluorescence staining of the spleen tissues in LPS-injected animals showed that TNF-α signals were strongly induced through the red and white pulps including the marginal zone, but were significantly decreased by MAC treatment. Here again, a splenic neurectomy and a vagotomy resulted in elevated TNF-α signals (Fig 2B and 2C). Together, these results suggest that TNF-α induced in the spleen and the serum after LPS administration may be modulated by AS.

**Fig 3:**
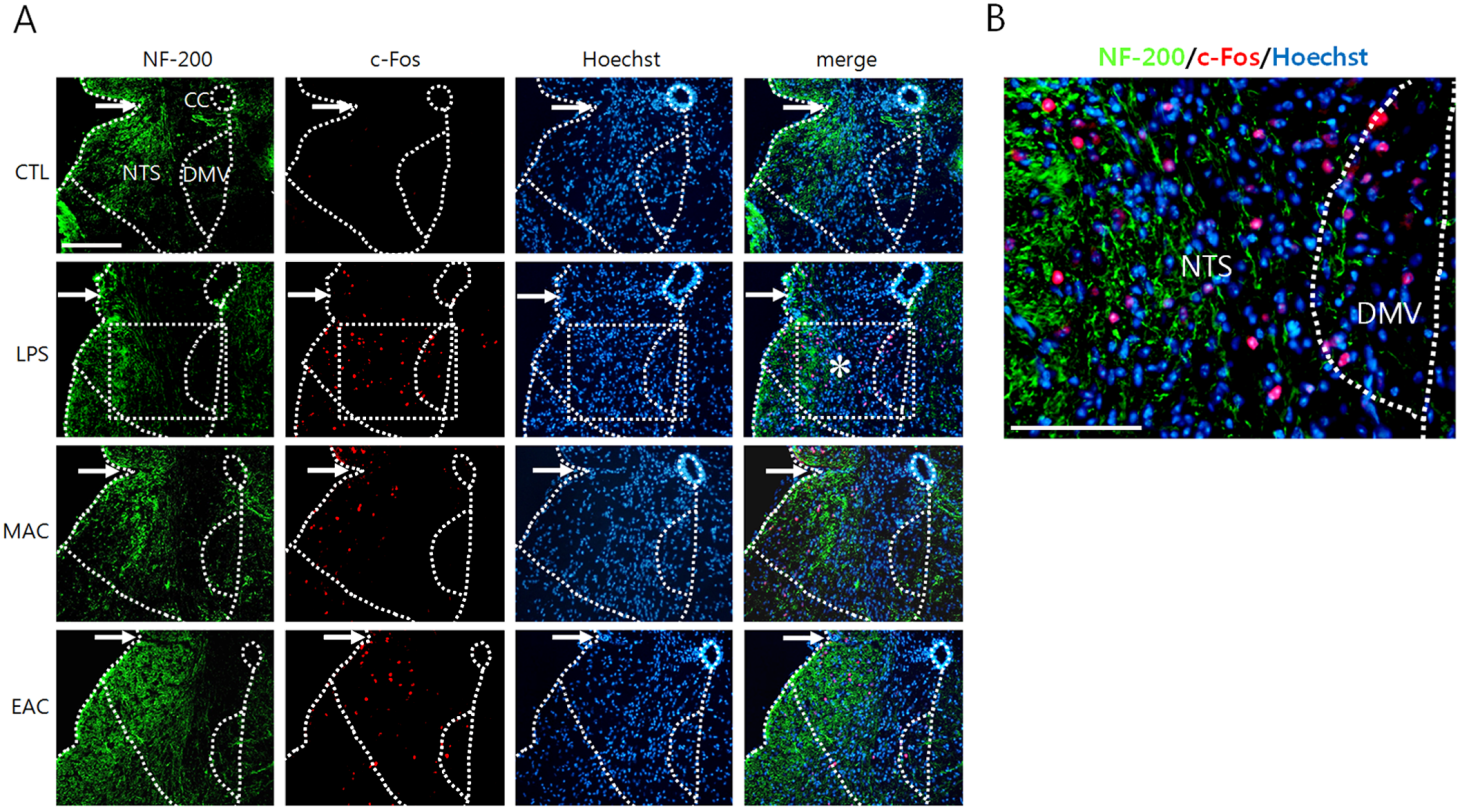
c-Fos induction in the DVC by AS and LPS administration. Following LPS injection (180 min later) or AS (90 min later), transverse sections through the caudal portion of the brainstem were used for immunofluorescence staining for NF-200 (green) and c-Fos (red) with Hoechst nuclear counterstaining (blue). An enlarged view (B) of the rectangular area in (asterisk in A) clearly reveals c-fos signals. Arrows in (A) denote the approximate NTS boundary between two hemispheres. cc: central canal. The scale bars in (A) and (B) are 100 μm.

**Fig 4:**
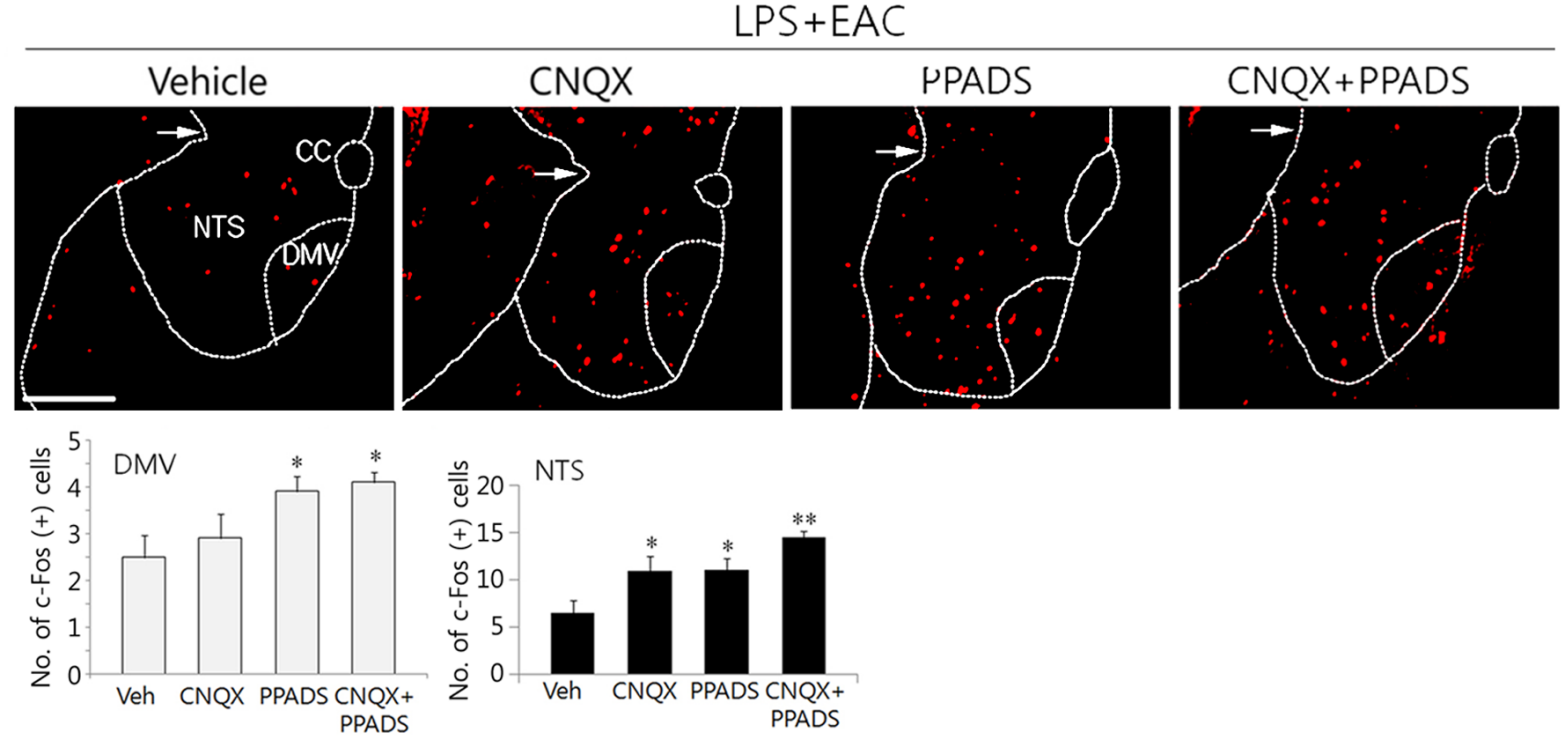
Regulation of c-Fos induction in the DVC by CNQX and PPADS. Following MAC and EAC in combination with focal administration of CNQX and/or PPADS into the DVC area, transverse sections through the caudal portion of the brainstem were subjected to immunofluoresence staining analyses for c-Fos production. Upper images are the representatives showing c-Fos signals in the brain sections and lower graphs show the quantitation of the protein signals in the field. Mean ± SEM (n = 4 independent experiment). *p<0.05 and **p<0.01 vs. vehicle control (one-way ANOVA). The scale bar is 100 μm.

Next, we examined c-Fos signals in the DVC area following LPS administration and AS. Previous studies have shown that TNF-α, which is peripherally produced after LPS administration, gains access to neurons in the DVC and induces c-Fos expression by modulating the neurotransmitter release from the vagal afferents [18]. Here, our data showed that LPS administration induced moderate levels of c-Fos signals in NTS and DMV neurons (Fig 3A and 3B). c-Fos was also induced by MAC, and its level was further increased when EAC used. To understand the physiological basis for acupuncture-mediated neuronal excitability, we examined c-Fos levels in the DVC neurons after focal administration of AMPA receptor blocker CNQX and/or purinergic receptor antagonist PPADS following LPS administration and EAC. Here, PPADS treatment significantly increased c-Fos expression in both the NTS and the DMV areas (Fig 4). Some additive effects of co-treatment with two inhibitors were noted in NTS areas. However, a two-way factorial ANOVA on the c-Fos levels in the DMV and the NTS did not show any interaction between the CNQX and the PPADS treatments (df = 1, F = 0.141, p = 0.714 in the DMV; df = 1, F = 0.754, p = 0.402 in the NTS).

**Fig 5:**
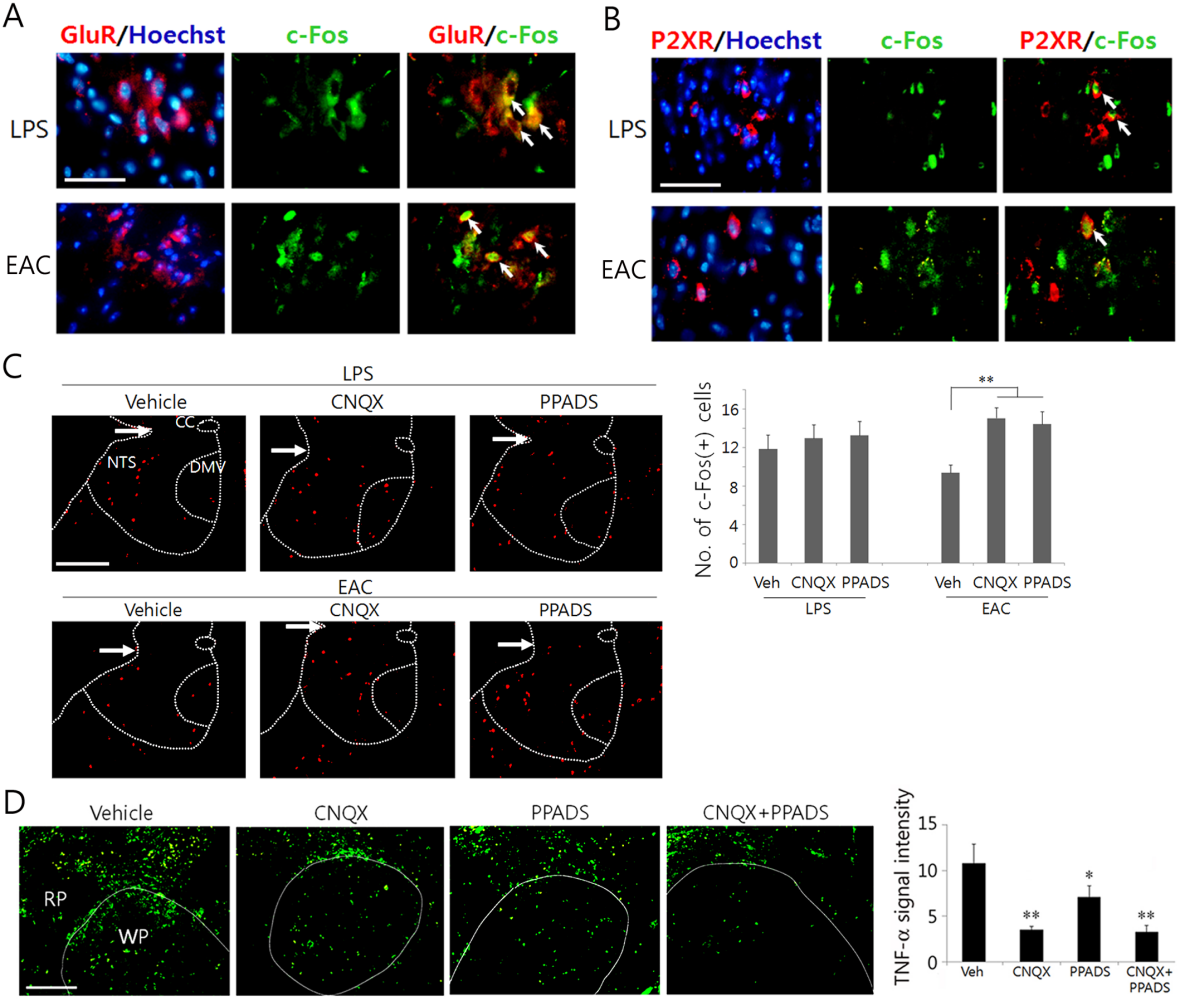
Identification of cells positive to glutaminergic and purinergic receptors and blockade effects of receptors on c-Fos and TNF-α production. Following LPS (180 min later) or EAC (90 min later), transverse sections through the caudal portion of the brainstem were used for immunofluorescence staining for GluR2/3 and c-Fos (A), P2X2 receptor (red) and c-Fos (green), and c-Fos. In (D), animals were treated with LPS and EAC, and drugs were also injected as indicated in the figure. Arrows in (A) and (B) denote the cells coexpressing GluR2/3 and c-Fos and P2X2 and c-Fos respectively. Arrows in (C) denote the approximate NTS boundary between two hemispheres. Quantifications of the protein signals are shown in (C) and (D). Mean ± SEM (n = 4 independent experiment). *p<0.05 and **p<0.01, *** vs. vehicle control (one-way ANOVA). The scale bars in (A-D) are 100 μm.

To determine whether LPS or EAC can selectively modulate the activity of AMPA receptors and purinergic receptors, we analyzed c-Fos signals in cells positive to GluR2/3 receptors and P2X2 receptors in the DVC area. Some of GluR2/3-positive cells showed c-Fos signals in LPS-administered or EAC-treated animals (arrow in Fig 5A). Similarly, there were P2X2 receptor-positive cells that expressed c-Fos protein in LPS-administered or EAC-treated animals (arrows in Fig 5B). To determine whether the inhibition of AMPA and purinergic receptors modulates the levels of c-Fos proteins induced by LPS or EAC, c-Fos was examined in the DVC area in the presence of CNQX or PPADS. In LPS-injected animals, c-Fos levels were not changed by CNQC and PPADS. However in EAC-stimulated animals, c-Fos levels in the NTS and DMV were significantly increased by CNQX and PPADS treatments (Fig 5C). Focal administration of CNQX and PPADS in the DVC significantly decreased TNF-α production in the spleen of the animals administered LPS and receiving EAC (Fig 5D). Co-treatment of CNQX and PPADS failed to show any effect of interaction on the splenic TNF-α levels (two-way factorial ANOVA: df = 1, F = 3.31, p = 0.094).

**Fig 6:**
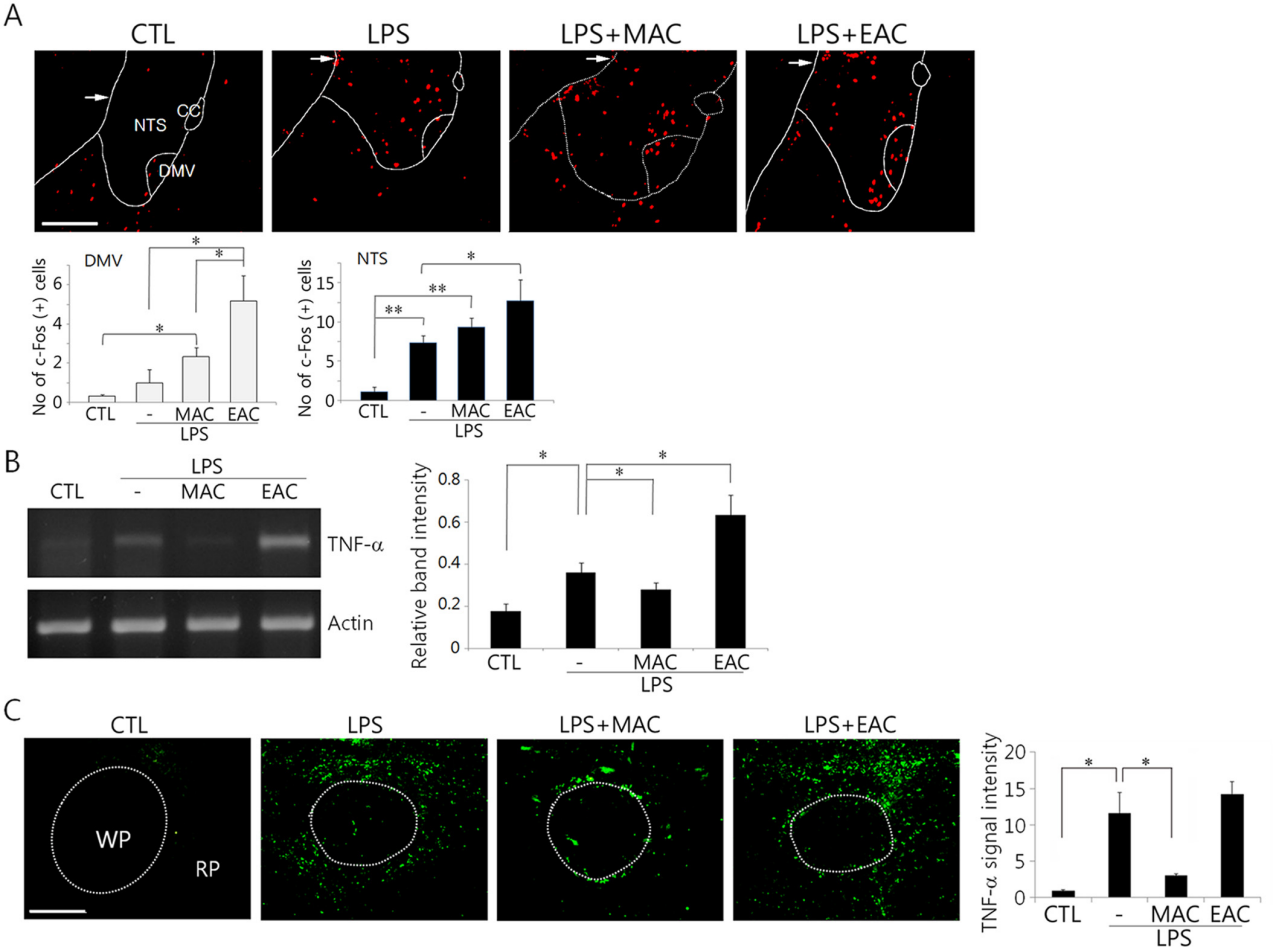
In vivo inductions of c-Fos and TNF-α in animals given LPS and AS without anesthesia. Sixty minutes after LPS injection, MAC or EAC was given at ST36 for 30 min. Animals were sacrificed 90 min after AS for analyses of the changes in both c-Fos protein signals in the transverse sections through the caudal portion of the brainstem (A) and the TNF-α mRNA and protein in the spleen (B and C, respectively). Quantitations of both the band intensity of splenic TNF-α mRNA relative to the actin control in (B) and the c-Fos and the TNF-α signals in (A) and (C) are shown in bar graphs (mean ± SEM, n = 4 independent experiments). *p<0.05 and **p<0.01 (one-way ANOVA). Arrows in (A) and (B) denote the approximate NTS boundary between two hemispheres. cc: central canal, WP: white pulp, RP: red pulp. CTL: non-treated control. The scale bars in (A) and (C) are 100 μm.

Anesthesia can attenuate TNF-α production in animals given LPS [11,19]. To examine whether the reduction in the TNF-α production in animals given AS under anesthesia was caused specifically by the AS, we investigated the effects of acupuncture therapy on c-Fos and TNF-α productions in unanesthetized animals. c-Fos was clearly induced in the DVC by LPS administration and further increased by MAC and EAC, though EAC was more effective than MAC (Fig 6A). TNF-α mRNA was induced in the spleens of mice that had been administered LPS and was significantly decreased by MAC. In contrast, EAC caused a further increase in TNF-α mRNA (Fig 6B). Similarly, MAC, but not EAC, was effective in downregulating the production of TNF-α protein in the spleen (Fig 6C).

## Discussion

The present study provides evidence that AS transmits signals into the vagus nerve and mediates anti-inflammatory responses in the spleen. Using an animal model of acute inflammation, we found that AS attenuated inflammatory responses as measured by TNF-α productions in the serum and the spleen. Removal of vagal and splenic nerves significantly abrogated the anti-inflammatory effects mediated by AS. Our study further shows that AS triggered neuronal activation in the DVC in which synaptic transmissions through both glutamate receptors and purinergic receptors are functionally involved.

The vagus nerve is the major parasympathetic nerve that is responsible for physiological regulation of most internal organs. The afferent component in the vagus nerve transmits sensory information from the cardiopulmonary and gastrointestinal organs to the solitary nucleus to communicate with the efferent components in the DMV and the nucleus ambiguous that travel back to the target organs. Growing bodies of evidence indicate that vagus nerve activity is important not only for homeostatic regulation of internal organs but also for the regulation of pathologic inflammatory reactions; thus, the vagus nerve acts as a bridge between the neural and immune systems [14,20]. Notably, VNS can activate the α7 nicotinic acetylcholine receptor on the macrophages in the spleen via splenic adrenergic inputs to T cells [21]. This ‘cholinergic anti-inflammatory reflex’ appears to control several inflammatory diseases such as rheumatoid arthritis, diabetes, and obesity [7,22]. Vagal activity has been previously implicated as a possible mediator of acupuncture therapy [3], and given the fact that, in principle, acupuncture works by coordinating interconnected body functions, exploring autonomic activity as the therapeutic basis of acupuncture is tempting.

Here, we found that TNF-α levels in the spleen as well as in the serum were decreased by AS in LPS-administered animals, but were elevated by a splenic neurectomy and a vagotomy. These results indicate that AS may activate the splenic nerve via vagus nerve activity to induce anti-inflammatory responses in the macrophages of the spleen. Having noted some previous reports indicating downregulation of TNF-α production by anesthetics [19,23], we performed additional experiments using unanesthetized animals to determine AS-mediated neural activity and inflammatory responses. While the data confirmed attenuation of TNF-α production by MAC, EAC further elevated TNF-α levels, raising the possibility that electrical inputs to the conscious brain may provoke inflammatory responses.

While our data clearly showed downregulation of splenic TNF-α production by AS, one recent paper reported that decreases in serum TNF-α level after EAC at ST36 were not affected by a splenectomy [7]. Here, the activity of the vagus nerve due to EAC was shown to induce dopamine production from the adrenal gland, which inhibited TNF-α production. However, in this study, the effects of EAC on TNF-α production were not investigated directly in the spleen tissue. Furthermore, subtle differences in the acupoints used, subject’s status, and the acupuncture procedure, such as MAC, EAC, and pharmacopuncture, may produce variability in the excitabilities among individual axons in the nerves and lead to different physiological consequences in the target organs [24].

Another issue that needs to be considered in relation to autonomic modulation of splenic TNF-α production is that the vagal branch to the spleen has not been clearly characterized so far [25–27], although a suggestion has been made that the celiac ganglion receives vagal inputs in addition to splanchnic, sympathetic inputs [28]. A proposal has been made that primary somatic afferents from the skin, muscles, and joints make synaptic connections to sympathetic preganglionic neurons in the spinal cord [2]. Here, our data showing that a vagotomy was less effective in negating the inhibition of TNF-α production in the spleen and serum by acupuncture compared to a splenic neurectomy suggest the possible involvement of sympathetic inputs in regulating splenic TNF-α production by acupuncture. Thus, in mediating AS, besides anti-inflammatory regulation via vagal efferent, a possibility of a splanchnic anti-inflammatory reflex cannot be ruled out [16,29].

According to our data, MAC, though its effect was milder than that of EAC, induced moderate levels of c-Fos expression in NTS and DMV neurons, indicating acupuncture-specific neuronal responses. As far as the sensory modalities, such as stretching, discriminative touch, and pricking, that can be elicited by AS are concerned [30], their neural pathway to DVC neurons in the brain stem remain elusive. According to a previous report [7], ST36 stimulation and sciatic nerve stimulation generate the same anti-inflammatory reactions. Sciatic afferents synaptically relay somatosensory information into the spinal cord, and ascending spinal neurons have axonal projections to NTS neurons [31–33]. Thus, AS at ST36 may reach NTS neurons through secondary or higher-order ascending spinal pathways.

Then, how would AS signals be transmitted to DVC neurons and activate them? Glutamate is a primary neurotransmitter that mediates excitatory signals in neurons of the DVC including vagal afferent fibers, and an involvement of GABAergic inhibitory neurons in the DVC were previously described in relation to the vago-vagal gastric reflex [34]. Interestingly, astrocytes in the NTS area respond to glutamate neurotransmitters via AMPA receptors leading to increased intracellular Ca^2+^ levels [35]. Astrocytes that bind to glutamate and ATP via AMPA/NMDA glutamate receptors and P2X purinergic receptors, respectively, are known to take part in neuron-glia interactions by releasing excitatory and inhibitory gliotransmitters to neighboring neurons [36]. Given the notion that the role of purinergic signaling has been proposed in mediating the peripheral acupuncture signals [37,38], it is tempting to explore the possibility of purinergic signaling in the DVC neural circuit to integrate acupuncture signals. Thus, we examined the involvement of glutaminergic and purinergic neurons in mediating peripheral acupuncture signals. Our data showed that the treatment with inhibitors of the AMPA receptor and the purinergic receptor upregulated c-Fos signals induced by EAC in the DVC neurons and led to decreased TNF-α signals in the spleen. One possible explanation for this observation would be that the neurons or astrocyets responding to acupuncture signals through the purinergic receptors and glutaminergic receptors might be present in the DVC and transmit inhibitory signals to NTS neurons and DMV neurons. Our data showed that EAC activated neurons or glial cells expressing glutaminergic receptors and purinergic receptors in the DVC, as evidenced by c-Fos signaling in AMPA- and P2X-positive cells. Consequently, vagal modulation of inflammatory reactions by acupuncture may be augmented by a disinhibition of synaptic transmission in the DVC circuit.

Given that ST36 is one of the most widely used acupoints for clinical therapy, acupuncture treatment at ST36 has been frequently investigated in animal experiments. Previous studies reported that acupuncture at ST36 was involved in regulating gastric motility and hyperalgesia associated with irritable bowel syndrome [39–41]. Molecular analysis revealed that ST36 stimulation in mice induced the releases of adenosine and pain-regulating receptors [38]. Whether combined stimulation at two or more acupoints, which is generally performed in clinical acupuncture therapy, may trigger a corresponding, integrative neural activity is unknown. An electrophysiological analysis of AS-specific responses from the nerves or ganglia connected to specific organs may be a useful approach to exploring the neural correlates to acupuncture therapy.
